# Recurrent Odontogenic Keratocyst Mimicking Persistent Periapical Lesion in the Anterior Maxilla: A Case Report With 15‐year follow up

**DOI:** 10.1155/crid/5295200

**Published:** 2026-03-08

**Authors:** Sumaya O. Basudan

**Affiliations:** ^1^ Department of Restorative Dental Sciences, College of Dentistry, King Saud University, Riyadh, Saudi Arabia, ksu.edu.sa

**Keywords:** case report, misdiagnosis, odontogenic keratocyst, periapical lesion, recurrence

## Abstract

Odontogenic keratocysts (OKCs) are aggressive odontogenic cysts with a high recurrence rate, often presenting diagnostic challenges due to their ability to mimic common periapical pathologies. Although more prevalent in the mandible, maxillary OKCs, especially those resembling periapical lesions, are rare and prone to misdiagnosis. This report details the case of a patient who presented with persistent swelling and pus discharge from the anterior maxilla, initially misdiagnosed as a chronic apical abscess following root canal treatment. Despite multiple endodontic interventions, symptoms recurred. Clinical and radiographic examinations revealed a large periradicular lesion associated with Tooth #12 (FDI). Following endodontic surgery and soft tissue debridement, histopathological analysis revealed the lesion as an OKC. The patient initially remained asymptomatic postsurgery, but recurrence necessitated a maxillary resection 5 years later. Annual follow‐ups over the subsequent 10 years showed no further recurrence. This case underscores the critical importance of including OKCs in the differential diagnosis of persistent periapical lesions, particularly in atypical presentations or cases unresponsive to conventional endodontic therapy. Thorough diagnostic investigation, including histopathological analysis, is essential for accurate diagnosis and definitive surgical management to prevent recurrence and improve patient prognosis, as is the importance of long‐term follow‐up. Moreover, our discussion of the literature highlights that OKCs in the anterior jaws may not be as uncommon as thought, and therefore should be considered in the differential diagnosis of periapical lesions.

## 1. Introduction

Lesions in the periapical area are typically of endodontic origin. They are often caused by microbial infection and generally respond well to conventional endodontic treatment with reported success rates as high as 93% [1, 2]. However, nonendodontic lesions can also present in the periapical area, posing diagnostic challenges [3, 4]. In one study, periapical lesions from 93‐failed root canal treatment cases were examined histologically. They found that 72% were periradicular granulomas, 21.5% cysts, 4.3% abscesses, and 2.2% scar tissue. These lesions can share similar radiographic appearances, making differentiation difficult. Notably, cystic prevalence tends to increase with the size of the periapical lesion. However, cysts cannot be definitively identified radiographically based on size or the presence of a radiopaque border [4]. Such nonendodontic cases are unresponsive to conventional root canal treatment [5].

The probability of encountering nonendodontic lesions in periradicular tissues is approximately 8% [6]. The reported incidence of nonendodontic lesions mimicking periapical pathosis varies, ranging from 0.65% to 10.94% [3]. For instance, a Brazilian multicenter study found that 4.22% of clinically diagnosed periapical lesions were histopathologically nonendodontic [6], whereas another study found the incidence of nonendodontic lesions in periradicular tissues obtained from endodontic surgeries to be 1.09% [7]. A recent systematic review reported that common nonmalignant nonendodontic lesions in the periapical area include odontogenic and nonodontogenic cysts and tumors, such as dentigerous cysts, odontogenic keratocysts, nasopalatine duct cysts, ameloblastomas, and adenomatoid odontogenic tumors, indicating a clear possibility of misdiagnosis [3].

Odontogenic keratocysts (OKCs) are distinctive developmental cysts of odontogenic origin, known for their aggressive growth patterns and high recurrence rates. They frequently mimic other more common periapical pathologies, posing a diagnostic challenge for clinicians. Although OKCs are more commonly found in the mandible, their presentation in the maxilla, particularly mimicking a periapical lesion, is less frequent and can lead to misdiagnosis and delayed appropriate management [3, 8].

This case report describes a rare presentation of an OKC in the anterior maxilla, initially misdiagnosed as a persistent periapical lesion following root canal treatment and retreatment. Surgical intervention and subsequent histopathological analysis of the lesion indicated that it was an OKC. The report highlights the importance of accurate and comprehensive examination in a systematic manner, thorough diagnostic investigation, appropriate management, and lengthy follow‐up, as well as considering less common pathologies, such as OKCs, in atypical presentations of periapical lesions.

## 2. Case Presentation

### 2.1. History

A 34‐year‐old South Asian (Pakistani) female patient with noncontributory medical history (American Society of Anesthesiologists Physical Status Classification I) presented to the Endodontics Clinics at the College of Dentistry at King Saud University. She was referred from the undergraduate clinics when her symptoms persisted at the 6 months follow‐up visit. The patient described that “I have swelling and pus discharge from my upper left front tooth that will not stop, although I received several root canal treatments.” She had first noticed swelling and discharge 4 years ago and received root canal treatment in her home country. Her symptoms did not subside, and discharge persisted. Three and a half years later, she sought treatment at the undergraduate dental clinics of the Dental College of King Saud University, where she was diagnosed with previous root canal treatment and chronic apical abscess in Tooth #12 (FDI notation). Radiographic examination of periapical radiographs indicated a large periapical lesion and root canal filling of inadequate quality. Subsequently, she received nonsurgical root canal retreatment over two visits with application of calcium hydroxide as an intracanal medicament before obturation (Figure 1). At the six‐month follow‐up visit, she was still complaining of recurring swelling and discharge although the pain had subsided, and she was subsequently referred to the endodontic clinics for assessment and management.

Figure 1Periapical radiographs of Tooth 12 prior to referral to the endodontic clinics. (a) Periapical radiograph at the time of patient presentation to the undergraduate dental clinics showing a large radiolucent lesion related to Tooth 12. (b) Periapical radiograph of Tooth 12 after the retreatment procedure was done.

**Figure:**
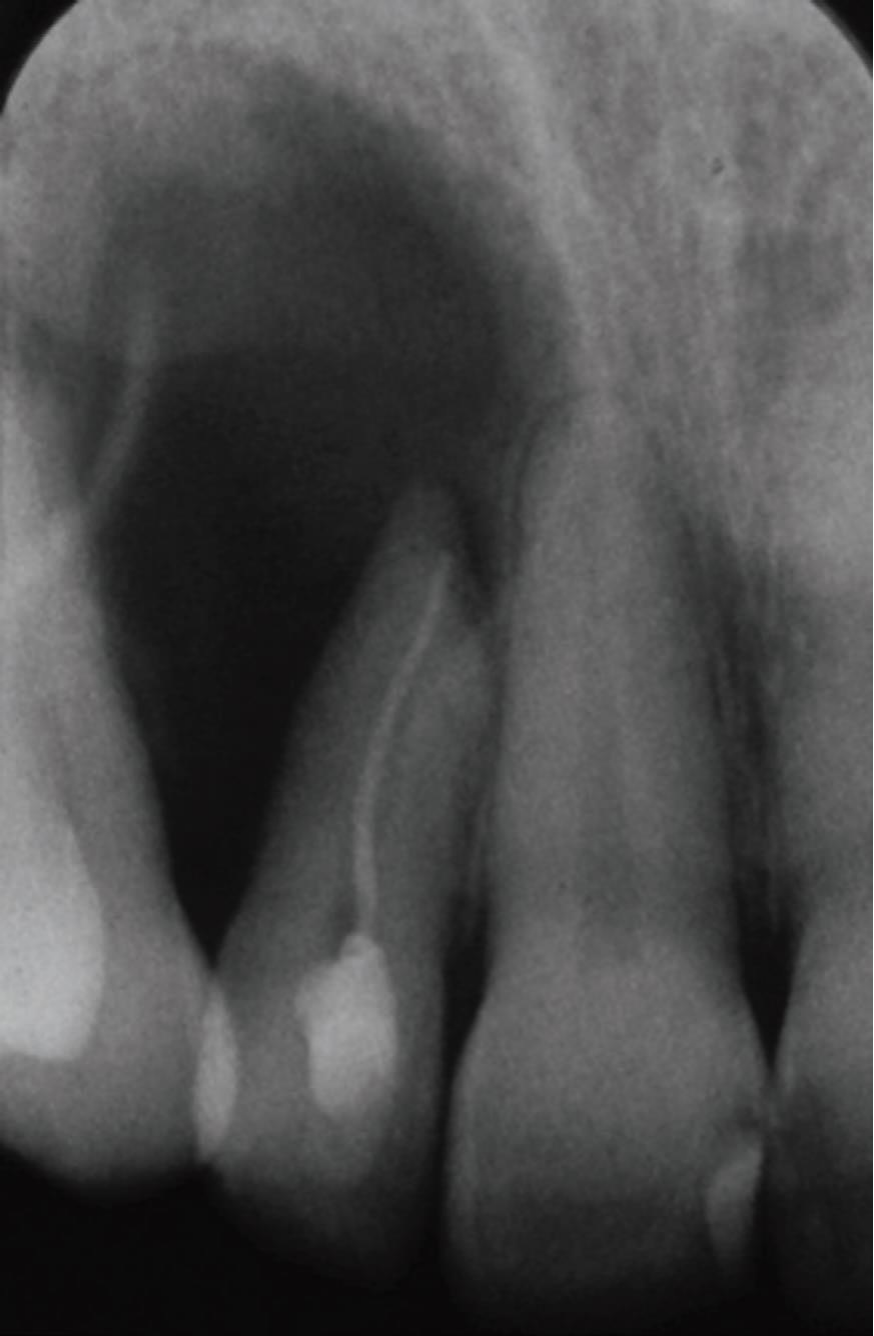
(a)

**Figure:**
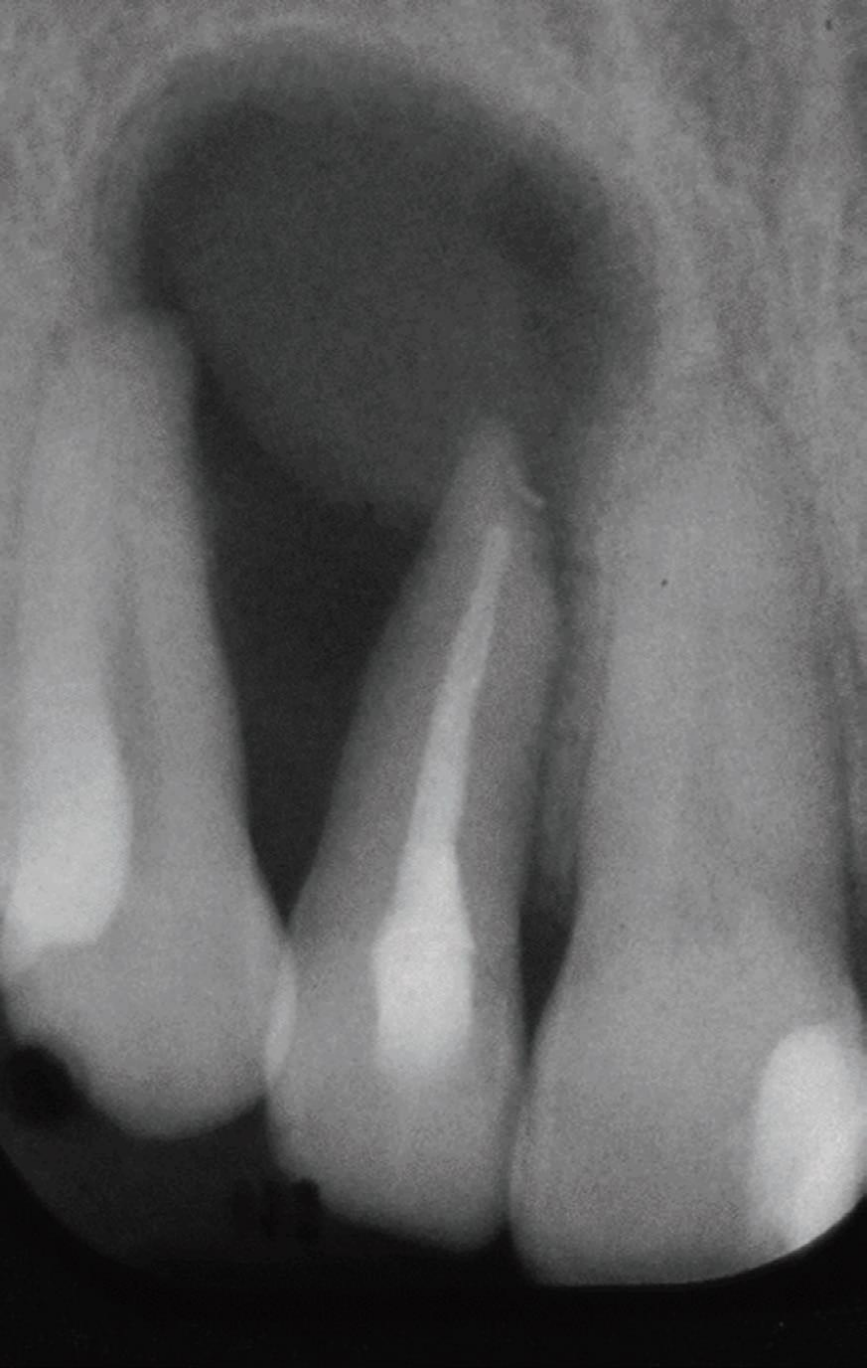
(b)

### 2.2. Examination

The patient was not complaining of pain, only recurrent swelling and discharge. Extraoral examination showed normal findings. Intraoral clinical examination revealed a palatal swelling. There was no buccal swelling or sinus tract. Tooth 12 had a large tooth‐colored restoration, Grade 2 mobility and an isolated 9‐mm pocket distolingually. Periapical radiographs showed a large periradicular lesion related to Tooth 12 involving the proximal area between Teeth 12 and 13 with displacement of the roots. The quality of the root canal treatment on Tooth 12 and its coronal composite resin restoration seemed satisfactory, and there was no need to repeat the endodontic retreatment procedure. The remaining maxillary anteriors were vital and responded positively to pulp sensibility testing.

### 2.3. Differential Diagnosis

Tooth 12 was endodontically diagnosed with previous endodontic treatment and chronic apical abscess. Given the persistence of symptoms despite retreatment, the differential diagnoses considered included nonhealing endodontic or nonendodontic etiologies, such as:

• Persistent endodontic lesion or abscess: The initial clinical presentation, but one that was unresponsive to conventional retreatment, suggesting a nonendodontic cause or a complex structural issue.
• Structural defect: A root fracture or crack, or an unaddressed developmental anatomical abnormality of the tooth, which could perpetuate the infection and persistent discharge.
• Radicular or lateral periodontal cyst: These were considered due to the large, well‐defined periradicular radiolucency. These lesions were also noted as possibly infected, contributing to the swelling and discharge.

### 2.4. Management

The case was presented to the patient and discussed in detail with her. Subsequently, agreement was reached towards a surgical approach to enucleate the lesion and to explore the underlying causes that were not clear clinically or radiographically. Endodontic surgery was initiated at the next visit, and loss of buccal and palatal plates was noted. Examination of the tooth surface under magnification by the dental operating microscope showed no signs of fractures or cracks or anatomical abnormalities on Tooth 12, even with staining by methylene blue dye. Soft tissue debridement was performed, and the excised tissue was sent for histopathological analysis.

### 2.5. Histopathology

The histopathology report of the soft tissue indicated an odontogenic keratocyst (Figure 2). Photomicrographs showed the pathognomonic histological features of OKCs, namely parakeratinized surface, thin epithelium of a cystic space, and hyperchromatic basal cells. The photomicrograph also shows the intense chronic inflammatory infiltrate seen in focal areas. It is important to know that whenever there is severe inflammation, the pathognomonic histological features of keratocysts are almost masked.

Figure 2Histopathology images of surgically excised tissue. (a) Low power magnification shows a cystic space lined by epithelium, and supported by thick fibrous connective tissue wall. This photomicrograph also shows that the lining is generally thin. (b) This photomicrograph shows focal thickening of the lining. (c) High power exhibits the distinctive features of odontogenic keratocyst, namely, corrugated parakeratinized surface, thin lining almost 5–6 cells thickness, prominent and hyperchromatic basal cells, and flat epithelium—connective tissue interface. (d) This photomicrograph shows the intense chronic inflammatory infiltrate seen in focal areas.

**Figure:**
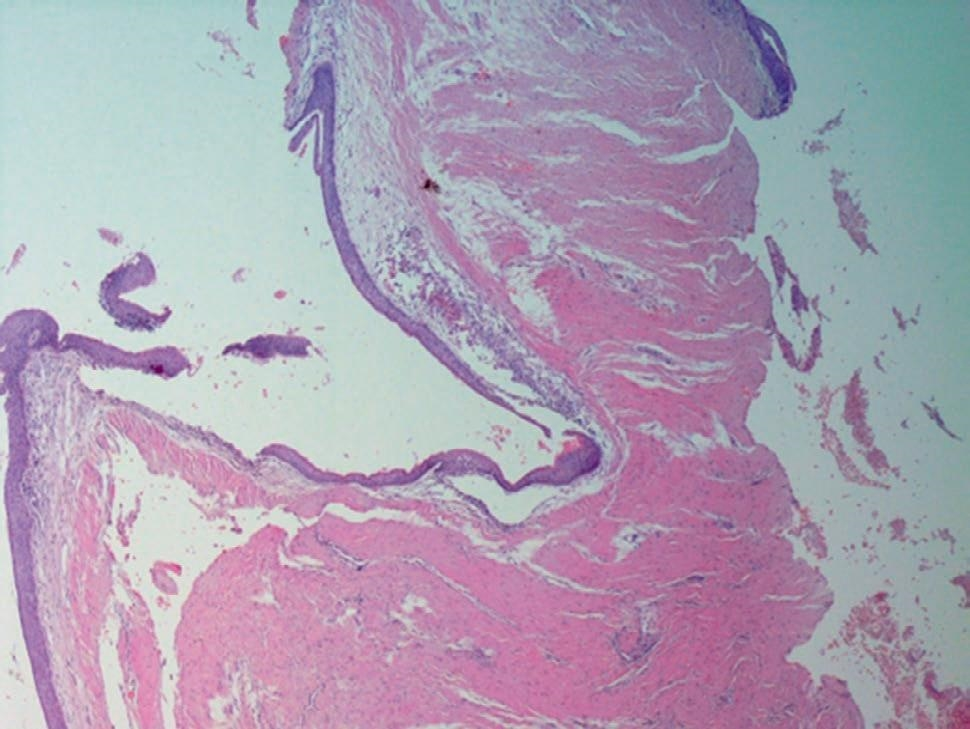
(a)

**Figure:**
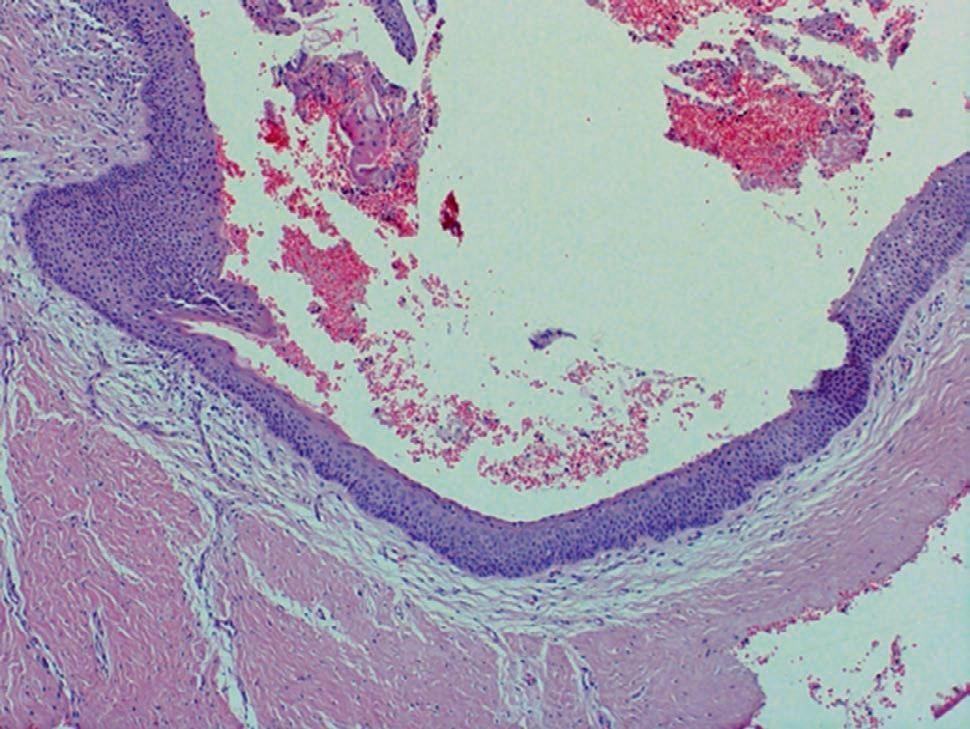
(b)

**Figure:**
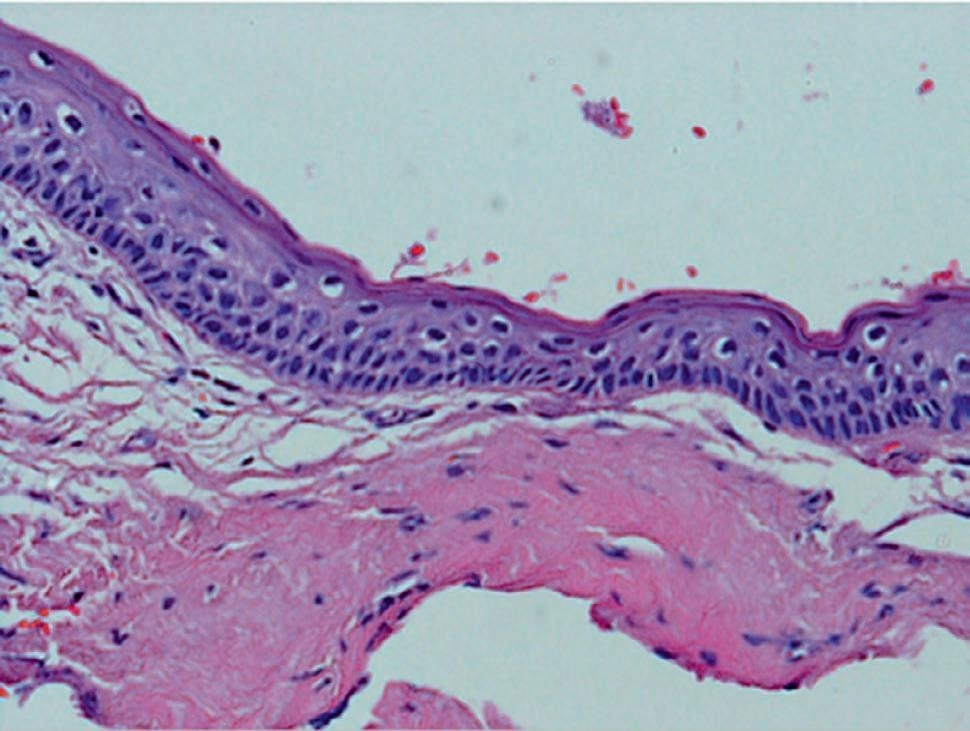
(c)

**Figure:**
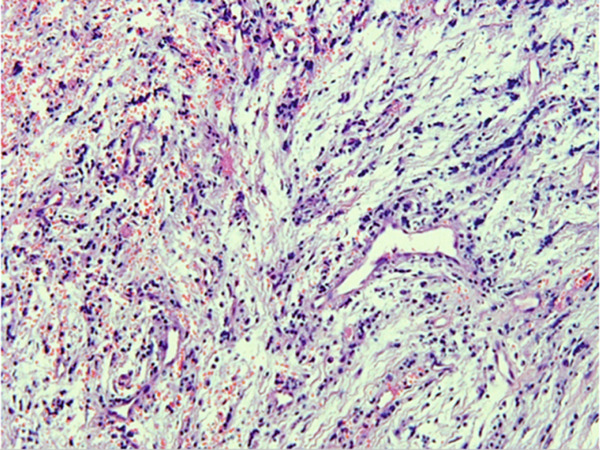
(d)

### 2.6. Follow‐Up

The patient was followed up 7 days postsurgically and was completely asymptomatic at 2 weeks. Follow‐up was continued at 4 and 8 weeks, 6 months, and then annually. At the 2‐year follow‐up, the patient′s symptoms were still absent; there was no recurrence of the cyst, and marked bone healing of the periapical area was observed. Mobility improved to Grade 1, and the periodontal pocket was reduced to 5 mm.

### 2.7. Recurrence

At the 5‐year follow‐up, the patient complained of “pain when I press,” pointing apically to Tooth 12. Clinical examination showed no swelling; Tooth 12 was not tender to percussion. No increase in mobility was noted, but there was a 7 mm pocket, and the area was positive to palpation. The periapical radiograph showed a remarkable increase in the size of the lesion (Figure 3). The lesion was expected to be a recurrence of the OKC, and the patient was accordingly referred to the oral and maxillofacial surgery department for appropriate management. With the diagnosis known beforehand, a more aggressive approach was attempted this time to prevent recurrence. Maxillary resection, including the removal of Teeth 12 and 13, was subsequently performed.

Figure 3Periapical radiographs of Tooth 12 following up the patient for the first 5 years. (a) Preoperative periapical radiograph at day of curettage surgery. (b) Eight‐week postsurgical recall periapical radiograph. (c) Recall at 2.5 years showing evidence of bone healing. (d) Recall at 4 years showing further demarcated bone healing. (e) Recall at 5 years showing the increased size of the lesion suggesting recurrence of OKC.

**Figure:**
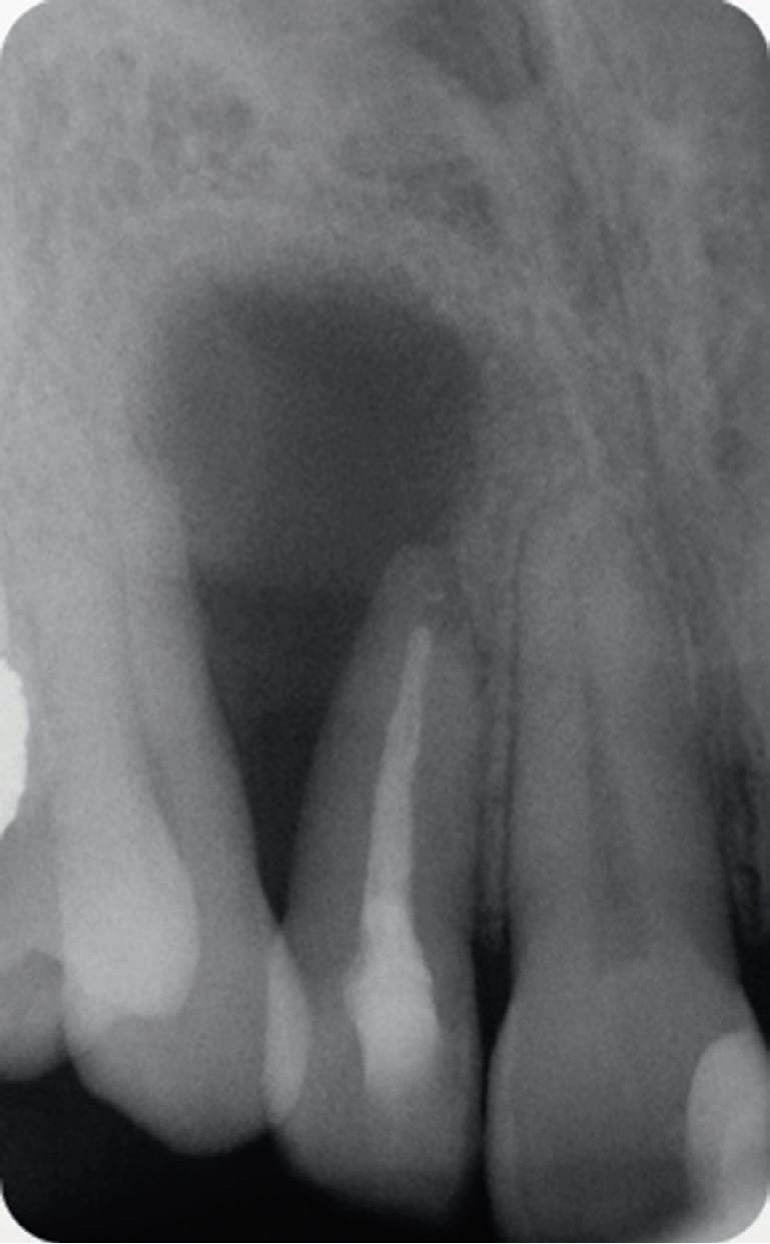
(a)

**Figure:**
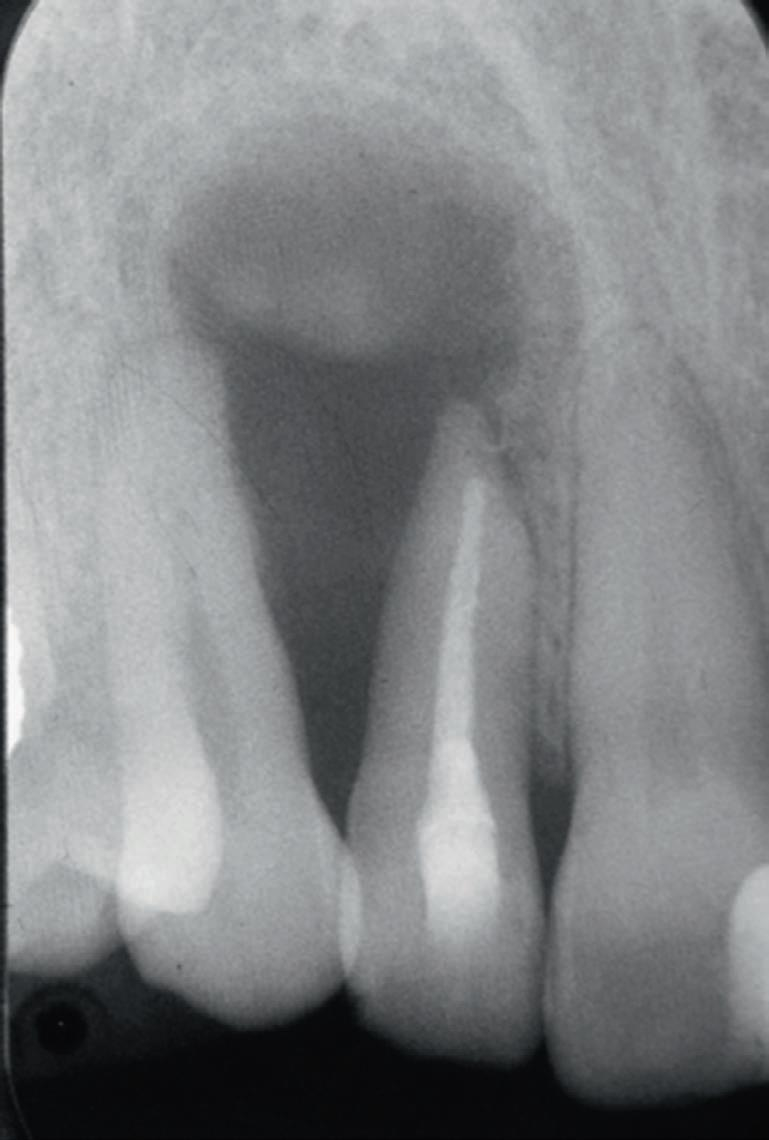
(b)

**Figure:**
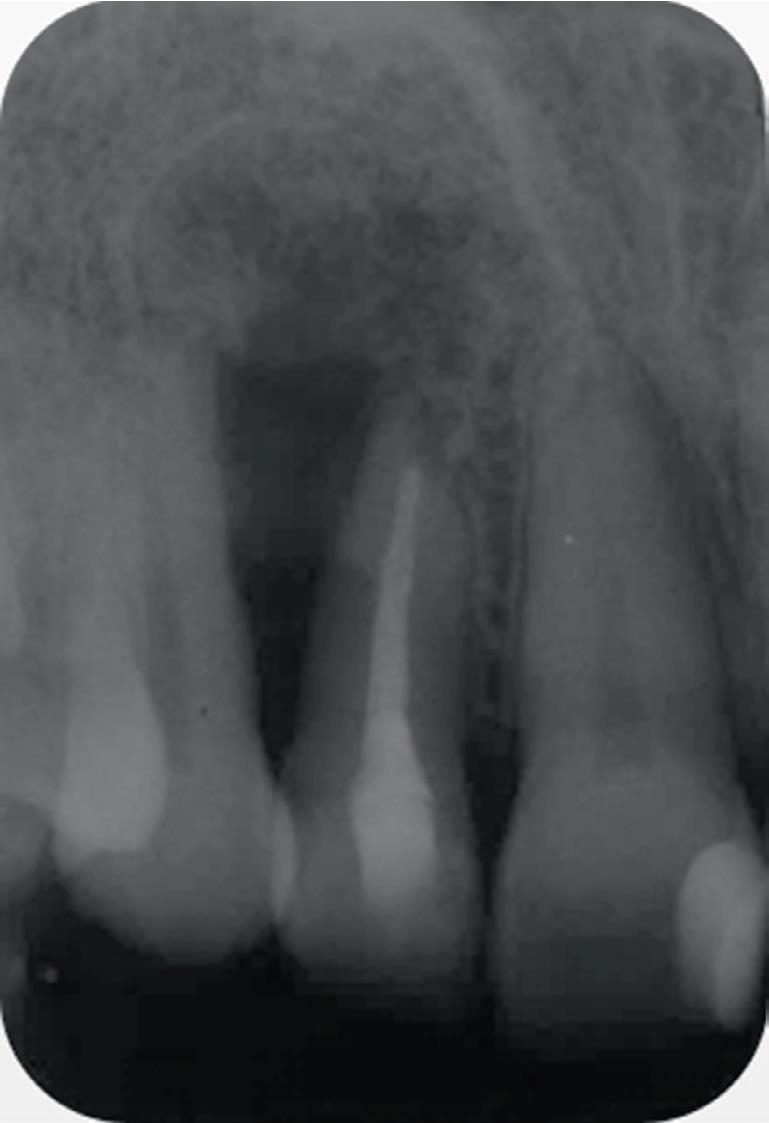
(c)

**Figure:**
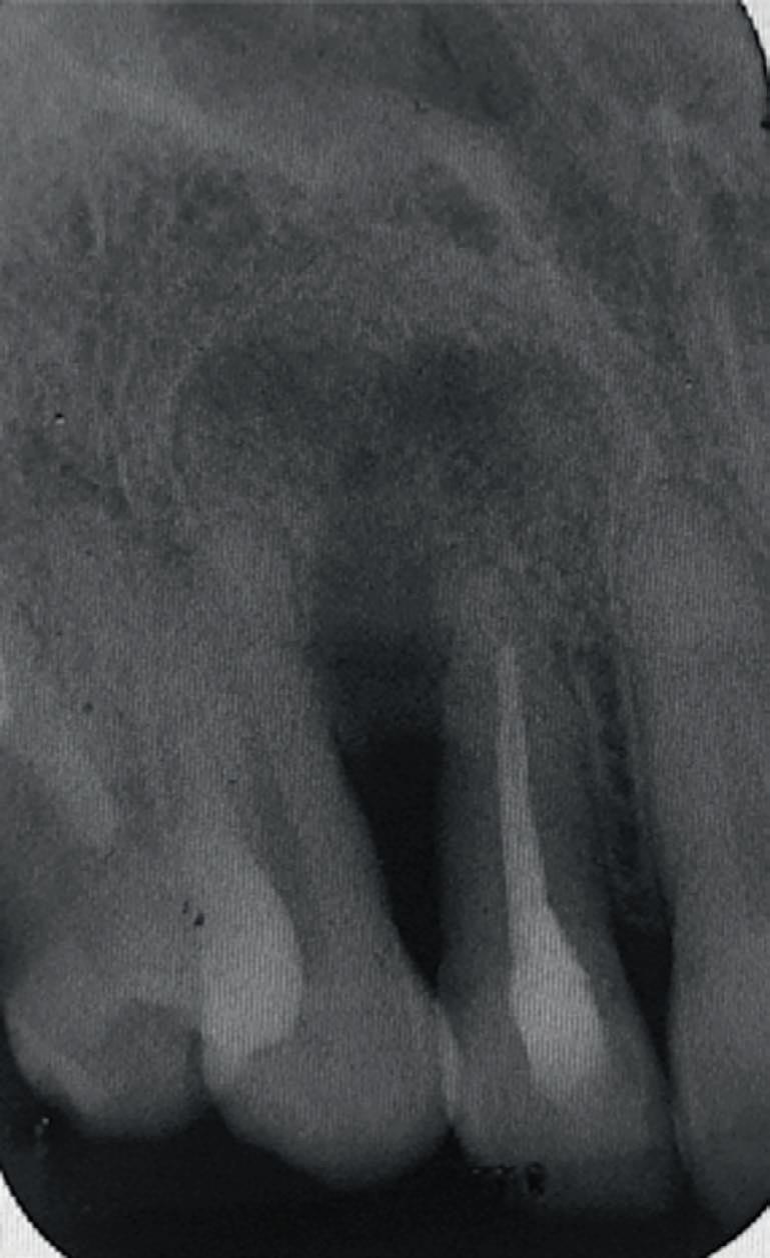
(d)

**Figure:**
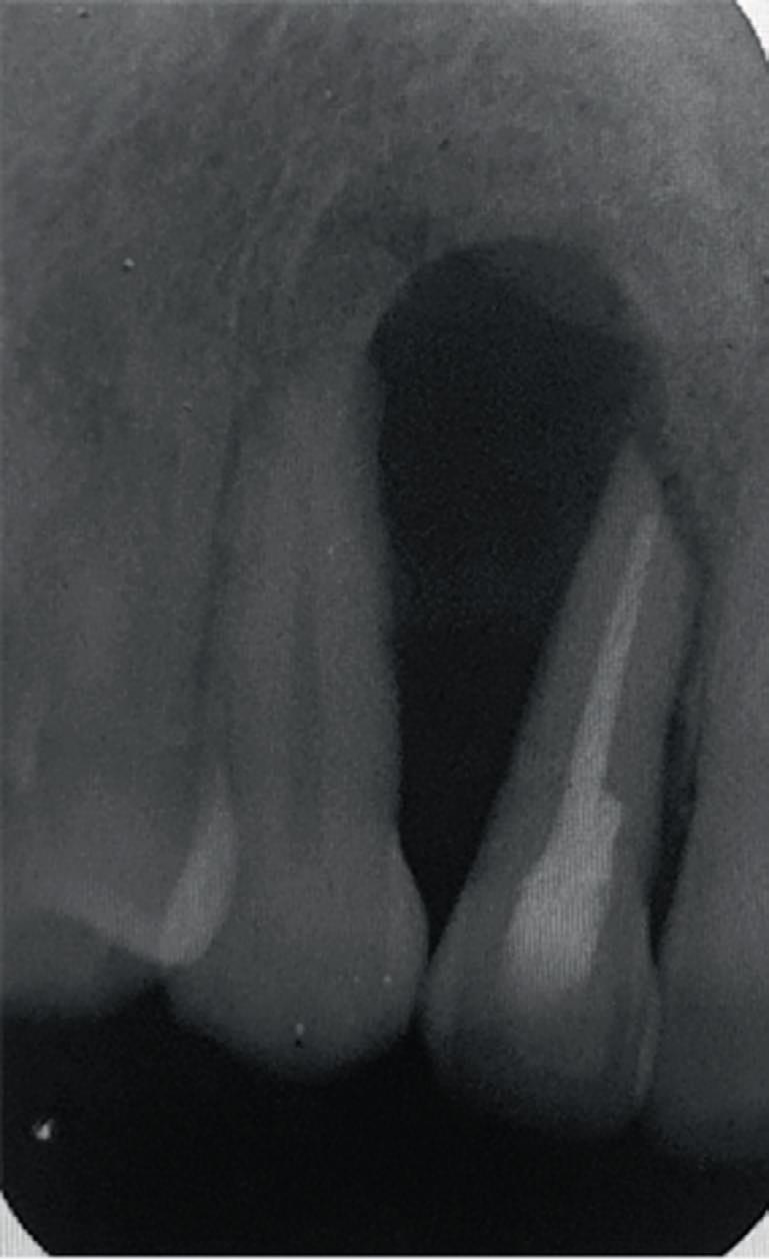
(e)

### 2.8. Follow‐Up After Resection

**Figure 5:**
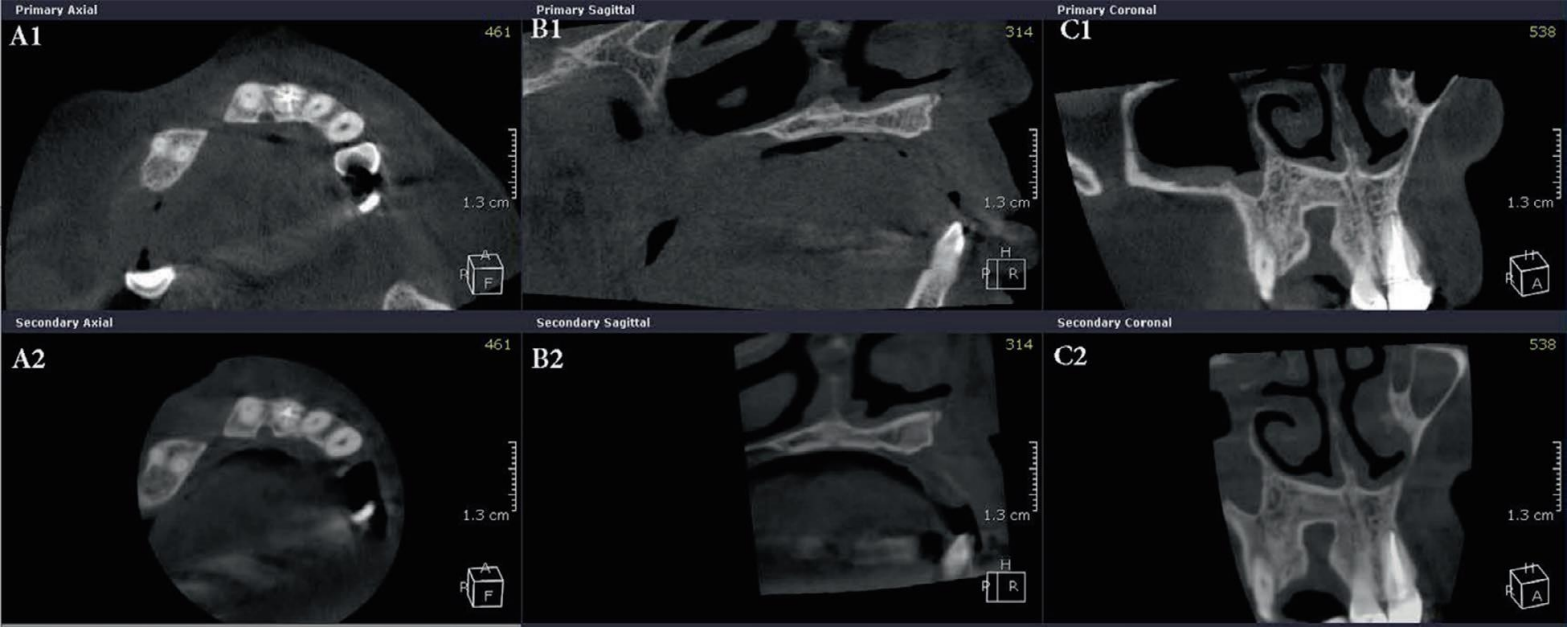
CBCT images show a well‐defined and corticated bony defect in the maxilla between Teeth 11 and 14. Taken from (A) axial, (B) sagittal, and (C) coronal sections. First row (A1), (B1), (C1) are images taken at the 5‐year follow‐up postsurgical resection. Second row images (A2), (B2), (C2) are taken at the 10‐year follow‐up postresection, indicating the stability of the lesion.

Annual follow‐up continued over the next 10 years and showed no recurrence of the patient′s signs and symptoms or the lesion. Figure 4 shows the panoramic radiographs prior to the resection surgery, and then at 10 years of follow‐up. Cone beam computed tomography (CBCT) images were also taken 5 and 10 years following the resection surgery (Figure 5). Images at the 5‐year follow‐up revealed a well‐defined and corticated bony defect in the maxilla between Teeth 11 and 14. The defect is noted to involve the buccal and lingual cortical plates and to extend from the crest of the ridge to near the floor of the nasal cavity. At the 10‐year follow‐up postresection, the image findings showed no significant radiographic signs of infection or lesion recurrence, indicating stability in the observed maxillary defect over the past 5 years postresection, with no significant changes that would warrant intervention. The patient is still kept under periodic observation.

Figure 4Panoramic radiographs before and after surgical resection of OKC related to Tooth 12. (a) Panoramic radiograph before maxillary resection. (b) Panoramic radiograph at 10‐year follow‐up post resection.

**Figure:**
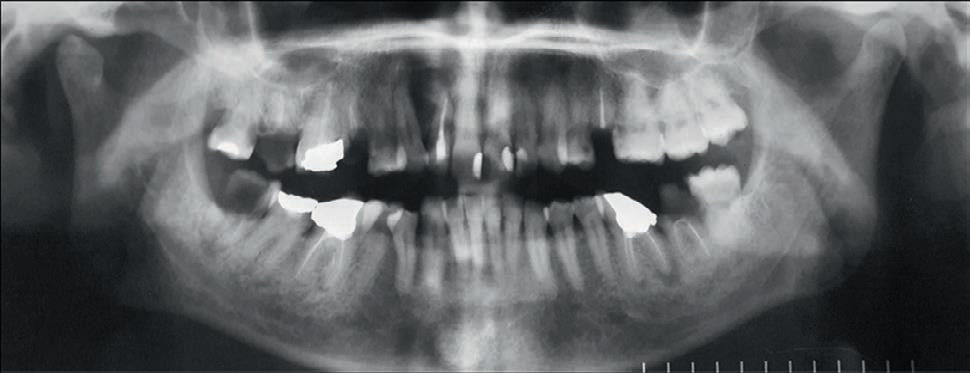
(a)

**Figure:**
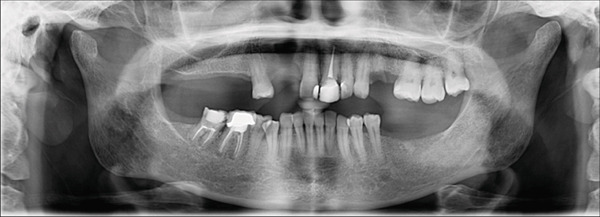
(b)

### 2.9. Outcome

Options for prosthodontic management to replace the missing teeth were discussed with the patient. She preferred a noninterventional option and requested a removable prosthodontic denture, which was fabricated and delivered. The patient was satisfied with the esthetic and functional outcomes. Moreover, at the 15‐year follow‐up visit, she continued to be asymptomatic without any pain or discharge.

## 3. Discussion

The OKC is a developmental odontogenic cyst derived from remnants of the dental lamina. It is important to accurately identify it, since it behaves more aggressively than other odontogenic cysts, has a higher recurrence rate, and might be associated with nevoid basal cell carcinoma syndrome [9]. Due to its aggressive nature, high relapse rate, invasiveness, presence of epithelial dysplasia, and gene mutations, the World Health Organization (WHO) classified it as a tumor between 2005 and 2017, but returned to its original classification as a cyst in 2017, as insufficient evidence was present for a tumor classification [10, 11]. The 2022 WHO Classification of Head and Neck Tumors continues to classify OKC as a cyst of the jaws [9, 12].

OKCs are the third most common cysts of the jaws, following radicular and dentigerous cysts, and account for about 10%–20% of odontogenic cysts [13]. The average age at diagnosis is 33.1 years, with incidence rates highest in the third decade, followed by the fourth, second, and fifth decades [9, 14]. Although some studies suggest a slight male predilection, others report no significant difference in prevalence between males and females [6, 9, 14].

Clinically, OKCs can be asymptomatic and discovered incidentally on radiographs, making early detection challenging. Their most common clinical presentation is swelling with or without purulent discharge. OKCs typically expand in an anteroposterior direction and may cause tooth displacement, as was first observed when the patient presented in this case. Radiographically, they may present as well‐defined, corticated, unilocular or multilocular radiolucent lesions that may cause jaw expansion [8, 15, 16]. CBCT imaging has the advantage of being more sensitive than periapical or panoramic radiographs, in addition to providing a more accurate and three‐dimensional view of the lesion [16]. In this presented case, a CBCT image was not taken preoperatively, as it was not yet available in the clinics at that time.

The majority of OKCs are found in the posterior mandible, particularly the ramus and third molar area [8, 14, 17]. However, cases in other locations, such as the mandibular premolar or anterior regions [18], or the anterior maxilla [19, 20], have been reported. A systematic review and meta‐analysis investigated the locations of OKCs and reported that 76% of OKCs occur in the mandible, of which 59% present in its posterior region, and 41% in the tooth‐bearing area. However, maxillary OKCs are more prevalent in the tooth‐bearing area (76.6%) rather than the posterior maxilla (24.5%) [8]. An earlier study supports this, identifying the maxillary canine area as the second most common site after the posterior mandible [21]. OKCs in the periradicular area of teeth often present as unilocular and may be associated with a higher recurrence rate in this region [16].

The most prevalent nonendodontic lesion in periapical areas, according to recent retrospective and systematic review studies, is the OKC [3]. A multicenter study of over 10,000 biopsies, initially suspected to be periapical lesions, found OKCs to be the most common of nonendodontic lesions in the periapical area (35%) [6]. Another center reported the most common nonendodontic lesion obtained from periapical biopsies during endodontic surgery was OKC too (25%) [7].

OKCs present a significant diagnostic challenge due to their clinical and radiographic resemblance to periapical lesions of endodontic origin. This misdiagnosis arises because OKCs can present as well‐defined radiolucent lesions surrounding teeth or in interradicular areas, or as asymptomatic gingival swellings located between roots of teeth [8]. Their presence in areas commonly associated with other benign cysts, such as lateral periodontal cysts (LPCs), particularly in the premolar and intercanine areas of both jaws, further complicates diagnosis. In one study, 182 of 742 (24.5%) histologically diagnosed OKCs were initially misdiagnosed as LPCs [15]. OKCs can also mimic radicular cysts or nasopalatine duct cysts when a small unilocular OKC appears in the anterior maxilla [3, 6].

Furthermore, they may be associated with teeth that are negative to sensibility tests, which typically suggests an endodontic etiology. Krongbarame et al. found that in teeth clinically diagnosed with pulpal necrosis, 2.36% of the biopsies showed that the periapical lesions were nonendodontic lesions. They also histologically identified endodontic lesions in a number of clinically vital cases, underscoring the potential for misdiagnosis based solely on sensibility tests [7].

Definitive diagnosis of OKC is based on histologic examination. Key histological features include parakeratinized stratified squamous epithelium lining the cystic cavity, a uniform thickness of 5–8 cell layers of epithelial lining, a corrugated appearance of the luminal surface, and columnar basal cells with palisading arrangement and reverse polarization of nuclei [9]. Although not definitive on their own, other diagnostic aids include radiographic features and aspiration biopsy. The presence of keratin flakes or a low protein level in aspirated fluid can be suggestive of OKC, whereas molecular analysis often reveals mutations in KRAS (Kirsten rat sarcoma virus gene) and activation of the MAPK (mitogen‐activated protein kinase) pathway, a cell signaling cascade regulating cell growth, which both are common findings that contribute to the aggressive nature of the lesion pathway in most cases [9, 15, 16].

The primary focus of OKC management is preventing recurrence, with common approaches including surgical excision and adjuvant therapies [22]. Surgical options include enucleation, the complete removal of the cystic lining, which can have a significant recurrence rate; and en bloc excision, which involves removing the cyst with a margin of surrounding bone and has shown no observed recurrence in some studies [14, 16]. Adjuvant therapies are often used alongside enucleation to reduce recurrence, such as Carnoy′s solution, a chemical cautery used to destroy residual epithelial cells or satellite cysts; cryotherapy, which involves freezing the cyst lining; peripheral ostectomy, the removal of a thin layer of bone surrounding the cyst cavity; and decompression, which aims to reduce the size of the cyst, but may leave satellite cysts, potentially leading to higher recurrence rates if not followed by definitive removal [14, 16, 23].

The high recurrence rate of OKCs is due to their thin, fragile epithelial lining and the presence of satellite cysts [16]. Their recurrence rate has been reported to be as high as 58.3% [14]. Although a recent systematic review calculated the mean recurrence to be around 16% [23], these varying rates are related to the management technique. Simple enucleation is associated with the highest recurrence rates of up to 43%, whereas resection with the lowest (2%) [14, 23]. The use of Carnoy′s solution significantly reduces recurrence; however, it is not appropriate for the tooth‐bearing area because it may damage the periodontium of teeth involved [8, 16].

Recurrence risk also increases with lesion size and multilocular forms. Titinchi′s review of OKC recurrence risk factors led to a point‐based calculation incorporating clinical, radiological, histopathological, and immunohistochemical features. Patients scoring less than two points are categorized as low risk, whereas those with more than six points are high risk. Based on age, cortical perforation, and tooth association with the lesion, the patient in this report scored nine points, indicating a high risk for recurrence. This stratification could aid clinicians in early identification of high‐risk cases, facilitating appropriate management and follow‐up [16].

Most recurrences (74.3%) occur within 5 years of the initial surgery, as was observed in this presented case, but around 20% can appear between 5 and 10 years postoperatively, and some have been observed even up to 21 years later [14, 16]. A follow‐up protocol of every year for 5 years, then every 2 years, for a 10‐year period, with regular periodic radiographic examinations is recommended for early detection, especially in cases of initial recurrence due to the tendency for repeated recurrence [8, 14].

Accurate diagnosis is essential for successful endodontic treatment, particularly when distinguishing between endodontic and nonendodontic lesions. Misdiagnosis, especially confusing OKCs with periapical lesions of endodontic origin, can lead to inappropriate treatment, delaying correct management and potentially exacerbating the condition through ongoing symptoms and bone destruction. This differentiation is crucial as OKCs require an aggressive surgical approach due to their high recurrence rate, unlike endodontic lesions, which typically respond to conservative root canal treatment. Clinicians must conduct a thorough and systematic clinical examination and aim to establish a definitive diagnosis before initiating treatment, thereby minimizing diagnostic errors and optimizing outcomes. Close follow‐up is also essential to ensure complete healing of the lesion. Whenever a periapical lesion exhibits aggressive characteristics, an unusually large size, or fails to heal, OKC should be considered in the differential diagnosis, regardless of the location of the lesion in the jaws. Given that OKCs in tooth‐bearing areas are often not diagnosed until histopathological analysis postsurgically, a biopsy can aid in establishing the correct pathological diagnosis and treatment course. Histologic examination is indispensable for differentiating endodontic from nonendodontic lesions, ensuring timely and appropriate management, and improving patient prognosis.

## 4. Conclusion

OKCs should be considered in the differential diagnosis of persistent periapical lesions, especially in atypical presentations or cases unresponsive to conventional endodontic therapy. The initial misdiagnosis and subsequent recurrence in this case highlight the aggressive nature of OKCs and support the need for a systematic approach: thorough diagnostic investigation, including histopathological analysis, definitive surgical management, and lengthy long‐term follow‐up are all paramount for achieving successful, recurrence‐free outcomes.

## Author Contributions

**Sumaya O. Basudan:** conceptualization, visualization, writing – original draft, editing, reviewing.

## Funding

No funding was received for this manuscript.

## Consent

Patient has consented to publishing this case report in this journal and has signed the consent agreement form. None of the details identifies the patient.

## Conflicts of Interest

The author declares no conflicts of interest.

## Data Availability

The data that support the findings of this study are available from the corresponding author upon reasonable request.
